# Supplementary material for: Prevalence of smoking restrictions and child exposure to secondhand smoke in cars and homes: a repeated cross-sectional survey of children aged 10–11 years in Wales
Source: BMJ Open. 2015 Jan 30;5(1):e006914. doi: 10.1136/bmjopen-2014-006914 (PMC4316441; doi:10.1136/bmjopen-2014-006914)
Supplement: Author's manuscript [file bmjopen-2014-006914.draft_revisions.pdf]

BMJ Open

Childhood second-hand smoke exposure in cars and homes: a repeated cross-sectional survey of 10-11 year old children in Wales

Journal:	*BMJ Open*
Manuscript ID:	bmjopen-2014-006914
Article Type:	Research
Date Submitted by the Author:	14-Oct-2014
Complete List of Authors:	Moore, Graham; Cardiff University, School of Social Sciences<br>Moore, L; University of Glasgow, MRC/CSO Social and Public Health Sciences Unit<br>Littlecott, Hannah; Cardiff University, School of Social Sciences<br>Ahmed, Nilufar; Cardiff University, School of Social Sciences<br>Lewis, Sophia; Cardiff University, School of Social Sciences<br>Sulley, Gillian<br>Jones, Elen<br>Holliday, Jo; Cardiff University, School of Social Sciences
**Primary Subject Heading**:	Public health
Secondary Subject Heading:	Smoking and tobacco
Keywords:	PUBLIC HEALTH, PREVENTIVE MEDICINE, SECONDHAND SMOKE

SCHOLARONE™
Manuscripts

For peer review only

Childhood second-hand smoke exposure in cars and homes: a repeated cross-sectional survey of 10-11 year old children in Wales

CORRESPONDING AUTHOR

Graham F Moore

Address: Centre for the Development and Evaluation of Complex Interventions for Public Health Improvement (DECIPHer), 1-3 Museum Place, Cardiff University, School of Social Sciences, Cardiff, CF10 3BD

Email: MooreG@cf.ac.uk

Telephone number: 02920 875360

CO-AUTHORS

Laurence Moore

MRC/CSO Social & Public Health Sciences Unit, University of Glasgow, UK

Hannah J Littlecott

Centre for the Development and Evaluation of Complex Interventions for Public Health Improvement (DECIPHer), Cardiff University, UK

Nilufar Ahmed

Centre for the Development and Evaluation of Complex Interventions for Public Health Improvement (DECIPHer), Cardiff University, UK

Sophia Lewis

School of Social Sciences, Cardiff University, UK

Gillian Sulley

Centre for the Development and Evaluation of Complex Interventions for Public Health Improvement (DECIPHer), Cardiff University, UK

Elen Jones

Centre for the Development and Evaluation of Complex Interventions for Public Health Improvement (DECIPHer), Cardiff University, UK

Jo Holliday

Centre for the Development and Evaluation of Complex Interventions for Public Health Improvement (DECIPHer), Cardiff University, UK

KEYWORDS

For peer review only

Secondhand smoke, public policy, prevention, children.

WORD COUNT

3699

For peer review only

ABSTRACT

Background

Surveys conducted immediately after legislation prohibiting smoking in public places showed small declines in childhood exposure to secondhand smoke (SHS) in cars and homes. Few studies have examined whether these declines continued in the longer term. This study examines children's exposure to SHS in cars and homes in Wales in 2014, and changes since 2008 post-legislation surveys.

Methods

CHETS Wales was a repeated cross-sectional survey of 10-11 year old children within 75 primary schools, involving self-report questionnaires and saliva samples from 1600 children in 2007/08. A replication survey (CHETS Wales 2) was conducted in 2014.

Results

The percentage of children who reported that smoking was allowed in their family vehicle fell from 18% in 2008 to 9% in 2014 (OR=0.42; 95% CI=0.33 to 0.54). The percentage living in homes where smoking was allowed decreased from 37% to 26% (OR=0.30; 95% CI=0.20 to 0.43). Among children with a parent who smoked, 1 in 5 and 1 in 2 continued to report that smoking was allowed in their car and home. SHS exposure remained highest among children from poorer families.

Conclusions

Smoking in front of children has continued to decline. However, substantial numbers of children continue to be exposed to SHS in cars and homes, particularly among poorer families. A growing number of countries have implemented, or plan to implement, legislation banning smoking in cars carrying children. Attention is needed to the impact of this legislation on child health and health inequalities, and to further reducing smoking in the home.

For peer review only

STRENGTHS AND LIMITATIONS

- The study reports findings from a survey of a large (n=1601) nationally representative sample of 10-11 year old children in Wales, replicating earlier surveys in 20007/8.
- More than two-thirds of schools taking part in 2007/8 were recruited in 2014, with remaining schools replaced by schools from the same area and with comparable socioeconomic status. Samples were comparable on all socio-demographic measures.
- Substantial differences in childhood exposure to secondhand smoke in cars and homes between 2008 and 2014 surveys can therefore confidently be said to represent change over time.
- The study is limited by reliance on self-report measures of exposure to secondhand smoke in cars and homes, though measures are validated against cotinine data collected in 2007/8.
- It is also not possible to make causal attributions regarding how changes in exposure observed over time came about.

For peer review only

BACKGROUND

The dangers of secondhand smoke (SHS, or passive smoking) are now well established.[1 2] Indeed, the World Health Organisation (WHO) state that that 'scientific evidence has unequivocally established that exposure to tobacco smoke causes death, disease and disability'.[3] Growing recognition of the dangers of SHS led many countries, including all UK countries, to implement legislation prohibiting smoking in enclosed public places and workplaces in the last decade; by 2011, an estimated 11% of the world's population lived in countries where smoking was prohibited in public spaces.[4]

In 2004, it was estimated that 61% of disease caused by SHS exposure worldwide was borne by children, whose developing lungs and rapid breathing rate make them particularly vulnerable to SHS.[5] Hence, while smoke-free legislation was implemented with the primary objective of protecting adults such as hospitality workers, impacts on childhood SHS received significant international scrutiny. The case against legislation made by its opponents centred on arguments that banning smoking in public spaces would displace smoking into the home. Some evidence to support this claim was reported in Hong Kong[6] and the USA.[7] However, studies in all UK countries contradicted the displacement hypothesis. Increases in the adoption of voluntary home smoking restrictions were reported in Scotland[8 9] and England.[10] While in Wales the proportion of homes with full smoking restrictions did not change significantly,[11] fewer children reported that parents smoked inside the home after legislation.[12] Indeed, a growing body of international evidence indicates that smoke-free legislation was, in most cases, followed by increases in voluntary restrictions on smoking in private spaces.[13 14]

For peer review only

While the growing de-normalisation of smoking among children reflected by these trends is welcome, declines in childhood SHS exposure immediately after legislation primarily benefited groups who were at relatively low risk prior to legislation. Significant declines occurred primarily among children of non-smokers[15 16] and from more affluent families.[11 12] Substantial percentages of children continued to report exposure to SHS in homes and cars. In Wales for example, 1 in 5 children reported that smoking was allowed in their family car, while more than a third reported living in homes where smoking was allowed.[11] All measures of exposure to SHS in homes and cars indicated that, before and after legislation, exposure was particularly prevalent among children from poorer families.[11]

Debates regarding how to safeguard children from the dangers of SHS, and address the role of SHS in the intergenerational reproduction of socioeconomic inequalities, have therefore moved toward attempts to reduce smoking in cars and homes. Due to the private nature of these spaces, regulation of behaviour is often regarded as an invasion of privacy. Hence, legislation will often only be considered where efforts to achieve change via voluntary means have not fully addressed the problem. In particular, while homes remain children's main source of SHS exposure, some have argued that only the in the most authoritarian of states would legislation around smoking in the home be acceptable.[17] Hence, efforts to promote smoke-free homes remain focused on voluntary rather than legislative means.

However, cars represent a space in which behaviours are already heavily regulated, hence occupying an intermediate space between public and private.[17] Furthermore, while children are likely to spend less time exposed to SHS inside cars than inside homes, the small and enclosed nature of vehicles means that SHS exposure is likely to be of an intense nature.[18] Hence, in a growing number of countries including parts of Australia, Canada and the USA[19],

For peer review only

bans have been introduced on smoking in cars carrying children. Recent surveys indicate that most adults[20-22] and children [23] think that smoking around children and in cars carrying children should be banned, while organisations including the British Medical Association[24] have called for a ban on smoking in all vehicles. More recently, a call was issued by 600 UK respiratory health professionals for MPs to back a ban on smoking in cars carrying children.[25]

In England, a House of Commons vote in 2014 gave ministers the power to introduce a ban on smoking in cars carrying children. In Wales, the Welsh Government have attempted to restrict smoking in cars via voluntary means, announcing the 'Fresh Start Wales' campaign in October 2011. This campaign comprised a range of marketing techniques through multimedia advertisements with the tagline 'Smoking in your car poisons your children', signposting to services that support quitting. The Welsh Government indicated that if insufficient voluntary changes were observed over the following 3 years, legislation would be considered, with the Children and Families Act of 2014 giving Welsh Ministers the authority to pass such legislation. This paper presents findings of a replication of the earlier CHETS Wales[16] surveys commissioned by the Welsh Government to assist with informing a decision on whether to proceed with legislation. It examines changes in children's exposure to smoke in cars and homes, whether socioeconomic patterning in these spaces has changed over time, and children's own attitudes towards a possible ban on smoking in cars. In summary, the paper addresses the following key research questions:

- Has the adoption of smoking restrictions in cars and homes increased in Wales from 2008 to 2014?
- Have socioeconomic inequalities narrowed, widened or remained the same?

For peer review only

- Are increases in smoking restrictions in private spaces reported by children of smokers?
- What are children's views on whether or not smoking in cars should be banned?

METHODS

Study design

CHETS Wales was a repeated cross-sectional study of Year 6 Welsh school children in 2007 and 2008. A replication study (CHETS Wales 2) was commissioned to assess changes in smoking in cars and other private spaces in 2014. Both were reviewed and approved by the Cardiff University School of Social Sciences Research Ethics Committee.

Sampling

CHETS Wales recruited a nationally representative sample of 75 state maintained primary schools across Wales. Schools were stratified according to high/low (cut off point identified as average entitlement across whole sample; 17.12%) free school meal entitlement (as a proxy for socioeconomic status) and Local Education Authority. Within each stratum, schools were selected on a probability proportional to school size. Where schools declined to participate, replacement schools were identified from within the same stratum. For CHETS Wales, target sample sizes were based on power to detect change in overall SHS exposure, assessed salivary cotinine. While CHETS Wales 2 was focused on reported SHS exposure in specific locations, hence using questionnaire data, it replicated the sampling methods used for CHETS Wales. The same schools who took part in CHETS Wales were approached where possible. Schools who declined or could not be contacted were replaced with another school sampled from the same stratum. Schools were paid £50 each for their time. Within each school, one Year 6 (age 10-11) class was randomly selected to participate, with all students in the class being involved.

For peer review only

Consent and data collection

Consent and data collection procedures for CHETS Wales are described in detail elsewhere.[16] These were replicated for CHETS Wales 2, with the exception that no saliva samples were collected. In brief, consent was sought from schools and parents, and assent from children. Schools signed a written agreement. An opt-out consent procedure was used for parental consent in the majority of schools, with a small number requesting use of opt-in consent. Children were also assured that their participation was voluntary and given the opportunity to opt-out on the day. In all years, data were collected over a ten week period between February to April in each year of collection. Data were collected in the classroom environment by trained staff. All staff were provided with a data collection protocol and given training in the DECIPHer centre to maximise standardisation of data collection procedures across the schools and data collection sweeps. Class teachers were asked to be present for disciplinary purposes, but not to intervene in the data collection in any other way unless asked to do so by the member of the research team.

Variables

Smoking in cars and the home

Children were asked 'Is smoking allowed in your family car, van or truck? ('yes', 'no', 'I don't know' or 'don't have a family car, van or truck') as well as 'While you were inside a car yesterday was anyone smoking there?'. Home smoking restrictions were assessed by asking children 'Is smoking allowed inside your home?' ('No, smoking is not allowed at all', 'smoking is allowed in certain areas only', 'smoking is allowed anywhere in our home', 'smoking is allowed only on special occasions in our home', 'I don't know'). Children were also asked 'While you were inside your home yesterday was anyone smoking there?'. Parental smoking in the home was assessed with the question 'Do any of the following

For peer review only

people smoke in the home?' in relation to i) father, ii) mother, iii) stepfather (or mother's partner), iv) stepmother (or father's partner) with response options 'smokes every day', 'smokes sometimes', 'does not smoke', 'I don't know', 'I don't have or see this person'. The parent was classified as smoking in the home if the child responded 'smokes every day' or 'smokes sometimes'. Children were categorized as having i) no parent figures who smoke in the home, ii) a father figure only who smokes in the home, iii) a mother figure only who smokes in the home, iv) two parent figures who smoke in the home.

Objectively measured secondhand smoke exposure

Salivary cotinine (a metabolite of nicotine) is a well-validated biomarker of SHS exposure in the previous 72 hours [26]. Anonymous samples were assayed using capillary gas chromatography with a detection limit of 0.1ng/ml. Saliva samples were collected in 2007 and 2008, but not 2014. Hence, they are used to indicate the validity of self-reports of smoking in cars and homes.

Attitudes to banning smoking in cars

In 2014, children's attitude to banning smoking in cars were assessed by asking children to circle (on a scale of 1-5) how much they agreed or disagreed with the following statements: 'There should be a complete ban on smoking in cars'; 'Smoking should be banned in cars carrying children under 16'.

Socioeconomic status

Children completed the Family Affluence Scale (FAS[27]), which generates a composite scale based on responses to questions on bedroom occupancy, car and computer ownership, and holidays.

Age

For peer review only

Children were asked to indicate the year and month of their birth on the smoking questionnaire. The month that the questionnaire was completed was recorded, and children's age in years calculated.

Statistical analysis

Descriptive statistics were used to examine the comparability of samples at 2007, 2008 and 2014 in terms of sex, age, socioeconomic status, family structure and child smoking status. For all key variables other than parental smoking in the home (6.0%), data were missing in less than 5% of cases. The validity of self-report items used to assess smoking in cars and homes was examined by presenting median and interquartile range cotinine values, as well as the percentage of children whose saliva samples contained detectable traces of cotinine, by reported exposure. Subsequently, frequencies and percentages of children who reported exposure to secondhand smoke in cars and homes were calculated for all three time-points. Significance of change from 2008 to 2014 was evaluated using logistic regression models adjusted for age and family affluence, with the year of data collection entered as the primary independent variable. Odds ratios therefore represent the odds of a child reporting exposure to SHS in the location specified in 2014 relative to 2008. To account for the clustered nature of the data sample, random terms for school were included in all models. These analyses were run twice: firstly with the entire sample, and secondly limited to children with at least one smoking parent. The above models were also used to examine socioeconomic inequality in smoke exposure in private spaces, through inclusion of FAS scores in the models, and testing of FAS by survey year interactions. For consistency with earlier analyses of CHETS Wales data, models including family affluence terms were limited to children living with one or both parent figures, although sensitivity analyses indicated that models which did or did not exclude children in other living arrangements gave consistent results.

For peer review only

RESULTS

Response rates

Response rates for CHETS Wales are reported in detail elsewhere[16]. In brief, 75 of 119 schools approached participated (63.0%) at both time-points, with child level response rates of 91.5% and 90.4% respectively. Of the 75 schools who participated in CHETS Wales, four could not be invited to participate in CHETS Wales 2 due to closure or change in status (i.e. no longer a mainstream school). Of the remaining schools, 51 participated. Forty-three further schools were invited to participate before the target of 75 schools was reached (overall response rate=65.8%). Of 1862 pupils within selected classes, completed questionnaires were obtained from 1601 (86.0%). In schools where opt-out consent procedures were followed (n=74 schools, 1810 pupils), 56 children were opted-out by parents, 35 children refused, and 141 were absent on the day of collection. Data were obtained from 1578 pupils (87.2%). One school requested opt in consent. Of the 52 eligible pupils in this school, consent was given for 23 children (44.2%), all of whom provided data.

Sample description

Pupil demographics at each time-point are presented in Table 1. There were no significant differences between time-points, with the exception of FAS scores, which were highest in 2014. However, this was explained entirely by widespread computer ownership in 2014, with FAS scores almost identical at all time-points where this item was removed. Hence, for analyses using FAS, this item is removed. FAS scores with or without computers were highly correlated (r=0.87). There were no significant demographic differences between children attending schools that did or did not participate at all 3 time-points.

For peer review only

Table 1. Sample descriptions by survey year. Figures are frequencies (and percentages) unless otherwise indicated

	2007 (n=1612)	2008 (n=1605)	2014 (n=1601)
Boys	778 (48.5)	792 (49.4)	797 (49.8)
Mean (SD) age	10.9 (0.4)	10.9 (0.4)	10.9 (0.4)
Mean (SD) FAS score	5.6 (1.9)	5.7 (1.9)	6.6 (1.9)
Mean (SD) FAS score without computers	3.9 (1.5)	3.9 (1.4)	3.9 (1.4)
Two parent families	1120 (69.5)	1089 (67.9)	1075 (66.5)
Step families	170 (10.6)	175 (10.9)	152 (9.4)
Single mother	263 (16.3)	273 (17.0)	263 (16.3)
Single father	18 (1.1)	23 (1.4)	32 (2.0)
Self-reported smokers	24 (1.5)	18 (1.1)	12 (0.7)

Validity of self-reported measures of SHS exposure

Median and interquartile range salivary cotinine values (using 2007-08 data), broken down by responses to self-report measures of exposure are presented in Table 2. In addition, percentages of children with cotinine above the limit of detection are presented. In all cases children who reported being exposed in homes or cars provided samples with substantially higher cotinine concentrations, and were substantially more likely to provide samples containing a detectable level of cotinine, than those who reported that they were not. Where limited to children who reported that smoking was allowed in their home, median cotinine concentrations were 7 times higher where children reported that smoking was also allowed in their car by comparison to those who said it was not (1.3ng/ml vs 0.2ng/ml), and twice as high for children who reported being in a car where someone was smoking the previous day versus those who did not (1.6ng/ml vs 0.8ng/ml). Hence, items on smoking in cars reflected differences in objectively measured SHS exposure which were not explained by the fact that most children who reported exposure to SHS in cars were also exposed to SHS in the home.

Table 2. Salivary cotinine concentrations by responses to self-report items on exposure to SHS in cars and homes

	Median (and inter-quartile range) salivary cotinine	Frequency and percentage cotinine above Limit of

For peer review only

		concentration (ng/ml)	Detection
Smoking allowed in car	No (n=1689)	<0.1 (<0.1 to 0.2)	594 (35.2)
	Yes (n=569)	1.1 (0.4 to 2.2)	526 (92.4)
	Don't know (n=424)	0.1 (<0.1 to 0.8)	235 (55.4)
	Don't own a car (n=211)	1.1 (0.2 to 2.7)	179 (84.8)
In a car where someone was smoking yesterday	No (n=2653)	<0.1 (<0.1 to 0.6)	1320 (49.8)
	Yes (n=196)	1.4 (0.7 to 2.9)	186 (94.9)
Parent figures smoke in the home	None (n=1781)	<0.1 (<0.1 to 0.1)	588 (33.0)
	Father (n=272)	0.5 (0.1 to 1.2)	225 (82.7)
	Mother (n=299)	1.2 (0.4 to 2.2)	274 (91.6)
	Both (n=406)	1.8 (1.0 to 3.0)	396 (96.3)
Smoking restrictions in the home	Full (n=1557)	<0.1 (<0.1 to 0.1)	484 (31.1)
	Partial (n=672)	0.5 (0.1 to 1.6)	534 (79.5)
	None (n=337)	1.7 (0.9 to 2.9)	319 (94.7)

Changes in exposure to SHS in private spaces

Table 3 indicates that smoking in cars has fallen substantially since 2008, with small declines between 2007 and 2008, but halving of exposure since. For example, in 2014, 9% of children (11% of those who reported that their family own a vehicle and that they know whether or not smoking is allowed in it) reported that smoking was allowed in it, a decline from 18% (23%) in 2008. Similar declines were observed among children of smokers, though 1 in 5 continued to report that smoking was allowed in their family vehicle. In 2014, 4% of all children, and 7% of children of smokers reported having been in a car where someone was smoking the previous day; a halving of exposure since 2008.

Table 3. Frequency (and percentage) of 10-11 year old children in Wales reporting smoking restrictions in car

		Smoking allowed in family car?				In car where someone smoking yesterday?
		Yes	No	Don't know	No car	
Whole sample	2007	327 (20.4)	926 (57.8)	231 (14.4)	118 (7.4)	107 (6.9)
	2008	288 (18.0)	965 (60.3)	234 (14.6)	114 (7.1)	107 (6.7)
	2014	141 (8.9)	1140 (71.7)	195 (12.3)	115 (7.2)	57 (3.6)

For peer review only

Children with a parent who smokes	2007	301 (38.6)	272 (34.9)	114 (14.6)	92 (11.8)	102 (13.5)
	2008	259 (34.8)	284 (38.2)	123 (16.5)	78 (10.4)	98 (13.3)
	2014	131 (19.6)	371 (55.5)	87 (13.0)	79 (11.8)	46 (7.0)

As indicated in Table 4, percentages of children living in 'smoke-free' homes (i.e. homes where smoking is not allowed at all) increased slightly between 2007 and 2008, though more markedly between 2008 and 2014. Similar changes were observed for children of smokers, among whom half reported living in a smoke free home in 2014, compared to 1 in 3 in 2008, while 1 in 11 lived in a home with no smoking restrictions, compared to 1 in 4 in 2008. Table 4 also indicates small declines in percentages of children reporting that one or more parent figures smoked, falling from 47% in 2007 to 40% in 2014. Larger declines were observed in percentages reporting that one or more parent figures smoked in the home. Figures for children with a parent who smoked indicate substantial reductions in the proportion of children of smokers whose parents smoked in the home, falling from 74% in 2007 to 71% in 2008 and to 52% in 2014. Hence, by 2014, almost half of children who reported that at least one parent figure smoked, reported that those parent figures did not smoke in the home.

Table 4. Frequency (and percentage) of 10-11 year old children in Wales reporting that parent figures smoke and levels of smoking restrictions in the home

	No smoking parent figure	Father smokes	Mother smokes	Both smoke
2007	825 (52.8)	230 (14.7)	187 (12.0)	322 (20.6)
2008	858 (55.5)	235 (15.2)	187 (12.1)	267 (17.3)
2014	929 (60.2)	211 (13.7)	164 (10.6)	240 (15.5)
	No parent figure smokes in home	Father smokes in home	Mother smokes in home	Both smoke in home
All children				
2007	973 (63.2)	148 (9.6)	161 (10.5)	258 (16.8)
2008	1009 (66.8)	144 (9.5)	164 (10.9)	194 (12.8)
2014	1153 (78.0)	93 (6.3)	91 (6.2)	141 (9.5)
Children with one or more parents who smoke				
2007	192 (25.7)	142 (19.0)	158 (21.2)	254 (34.1)
2008	201 (29.2)	138 (20.1)	159 (23.1)	190 (27.6)
2014	289 (47.7)	92 (15.2)	88 (14.5)	137 (22.6)
		Smoking in the home		

For peer review only

	Full restriction	Partial restriction	No restriction
All children			
2007	841 (59.1)	385 (27.1)	196 (13.8)
2008	883 (62.7)	361 (25.6)	164 (11.7)
2014	1041 (74.3)	303 (21.6)	57 (4.1)
Children with one or more parents who smoke			
2007	220 (32.0)	285 (41.5)	182 (26.5)
2008	218 (33.7)	278 (43.0)	151 (23.3)
2014	294 (51.0)	231 (40.0)	52 (9.0)

Table 5 presents odds ratios and 95% confidence intervals from logistic regression models, examining change over time from 2008-2014, and associations of socioeconomic status (FAS score) with smoking in private spaces. These analyses show that all markers of exposure to SHS in cars and homes decreased significantly from 2008 to 2014. These results were maintained when the sample was restricted to those children with at least one parent figure who smokes. The likelihood of a child reporting exposure to SHS was significantly lower for children from more affluent families in relation to all measures of exposure. There were no significant interactions between SES and survey year, with the exception of the percentage of children reporting being in a car the previous day where someone was smoking, for which socioeconomic inequalities narrowed significantly. For all remaining measures of SHS exposure, there were no significant reductions or increases in inequality.

Children's views on smoking in cars

Among the whole sample, 71.2% (n=1109) of children agreed that smoking should be banned in cars, with 76.4% (n=1191) agreeing that smoking should be banned in cars if children were present. Where limited to children who reported that smoking was allowed in their family vehicle, a small majority agreed that smoking should be banned in all cars (55.0%; n=77) while a larger majority (61.4%; n=86) agreed that smoking should be banned in cars when children are present.

For peer review only

Table 5. Odds ratios and 95% confidence intervals from logistic regression models examining associations of year of data collection and SES with exposure to smoke in private spaces

		Smoking allowed in cars (yes vs no)	Smoking in car yesterday	Smoking restriction in the home		Smoking in home yesterday	Parent figures smoke in the home		
				Partial	None		Father only	Mother only	Both parents
	All children								
	N	**2407**	**2987**	**2664**	**2664**	**2955**	**2836**	**2836**	**2836**
Model 1	Year	**0.42 (0.33 to 0.54)**	**0.52 (0.38 to 0.72)**	**0.70 (0.59 to 0.83)**	**0.30 (0.20 to 0.43)**	**0.44 (0.36 to 0.53)**	**0.54 (0.42 to 0.70)**	**0.48 (0.36 to 0.64)**	**0.65 (0.49 to 0.86)**
	FAS	**0.74 (0.68 to 0.80)**	0.92 (0.83 to 1.02)	**0.77 (0.72 to 0.83)**	**0.63 (0.57 to 0.71)**	**0.70 (0.65 to 0.75)**	**0.73 (0.67 to 0.81)**	**0.72 (0.65 to 0.79)**	**0.67 (0.62 to 0.73)**
Model 2	FAS*Year	1.14 (0.95 to 1.37)	**1.28 (1.01 to 1.60)**	1.00 (0.88 to 1.14)	1.05 (0.80 to 1.38)	1.06 (0.91 to 1.22)	0.94 (0.78 to 1.15)	1.06 (0.84 to 1.34)	1.05 (0.86 to 1.29)
	Children of smokers								
	N	**982**	**1303**	**1149**	**1149**	**1303**	**1217**	**1217**	**1217**
	Year	**0.41 (0.31 to 0.53)**	**0.49 (0.35 to 0.69)**	**0.59 (0.48 to 0.73)**	**0.26 (0.17 to 0.39)**	**0.41 (0.33 to 0.51)**	**0.45 (0.33 to 0.60)**	**0.38 (0.28 to 0.53)**	**0.52 (0.38 to 0.70)**
	FAS	**0.87 (0.79 to 0.97)**	1.07 (0.96 to 1.20)	**0.82 (0.75 to 0.88)**	**0.70 (0.61 to 0.80)**	**0.80 (0.75 to 0.87)**	**0.88 (0.78 to 0.98)**	**0.86 (0.77 to 0.95)**	**0.79 (0.71 to 0.88)**

*significant ORs highlighted in bold

For peer review only

DISCUSSION

The findings presented in this paper suggest that the de-normalisation of smoking in front of children in enclosed spaces observed immediately after introduction of smoke-free legislation has continued.[10] While it is not possible to make firm causal attributions, it is perhaps plausible that this represents a continuation of the effects of smoke-free legislation, and that evaluations included follow-up periods which were too short in duration to fully capture its impacts. The proportion of children who report that smoking is allowed in their family car has halved, while the percentage of children living in smoke-free homes has increased to almost 3 in 4. While in 2008 a clear majority of children who lived with a parent who smoked reported that smoking was allowed in their home,[11] half now report that their home is smoke free. It has also become increasingly rare to allow completely unrestricted smoking throughout the home. Overall, 1 in 25 children, including 1 in 11 children with a parent who smokes, report that smoking was allowed throughout their home; less than half the proportions observed in 2008. Hence, even among children who live with at least one smoking parent figure, a clear minority now report that smoking is allowed in their car, while it is no longer clearly the norm for smoking to be allowed in the home. Parents who smoke are increasingly choosing not to do so in enclosed places where their children are present.

However, while these trends are encouraging, the proportion of children who do still report exposure to SHS in cars and homes remains a significant concern. One in 5 children with a parent who smokes reports that smoking is allowed inside their car. Two in 5 report only partial restrictions on smoking in the home rather than full restrictions. Furthermore, smoking in private spaces continues to represent a mechanism in the intergenerational reproduction of health inequalities. While exposure declined across the socioeconomic spectrum, with no

evidence of widening inequality, SHS exposure continued to be significantly higher among children from poorer families.

While efforts to promote smoking restrictions in the home continue to do so through promoting voluntary change, a number of previous surveys have indicated that the majority of adults support the introduction of legislation banning on smoking in cars carrying children.[20-22] This study indicates support for such a ban from children themselves, with a large majority indicating that smoking in cars carrying children should not be allowed. Indeed, while fewer children who reported that smoking was allowed in their family car agreed with proposed legislation, a clear majority felt that smoking in cars carrying children should be banned.

Strengths of this study include its large nationally representative sample. The 2014 survey successfully recruited two-thirds of the schools who took part in the earlier CHETS Wales study, and achieved a sample with no significant demographic differences to the original sample. Hence, differences between survey years can be confidently attributed to change over time. The study relies upon self-reports of SHS exposure. However, while no saliva samples were collected in 2014, for all self-reported indicators of SHS exposure, objective indicators of SHS exposure were consistent with children's reports in 2007/08. Hence, reductions in self-reports of SHS exposure can be confidently assumed to reflect meaningful reductions in SHS exposure.

Partly informed by the key findings from this study, the Welsh Government announced that it will introduce legislation banning smoking in cars carrying children similar to that in place in parts of Canada, Australia and the USA,[19] citing the high proportion of children with parents

For peer review only

who smoke still exposed to smoke in cars. Further research is needed to understand the impacts of this legislation on childhood SHS exposure, health outcomes and health inequalities, including issues relating to enforcement and compliance. In addition, there is a need for sustained attention to understanding how to reduce smoking in the main location in which children continue to be exposed the SHS; the home. Further reducing childhood SHS exposure, while eliminating socioeconomic inequality, will likely require a combination of efforts to help parents to successfully quit smoking, and to encourage those who continue to smoke not to do so in the home.

ACKNOWLEDGEMENTS

We thank the Social Research and Information Division (Welsh Government) for support and advice; and Public Health Division (Welsh Government) for funding the study, and the schools and schoolchildren who participated in the study. We also thank Natalie Richards and Kim Sheppard for administrative assistance, Sophia Lewis for assistance with questionnaire design, and all fieldworkers who assisted with data collection.

CONTRIBUTORSHIP

GM JH and LM were investigators on the CHETS 2 study, and were responsible for study conception and design. The set-up and conduct of the survey were managed by NA, under the supervision of GM and JH. GM developed the paper plan, and led statistical analysis and drafting of the manuscript. HL contributed to acquisition of data, statistical analysis, drafting of the manuscript. SL assisted with study design, and wrote the first draft of a literature review which informed the background section. GS and EJ contributed to acquisition of data. All authors contributed to drafts of the full manuscript and approved the final draft.

For peer review only

FUNDING

The lead author is supported by an MRC Population Health Scientist Fellowship. The study was funded by the Public Health Division, Welsh Government. The work was undertaken with the support of The Centre for the Development and Evaluation of Complex Interventions for Public Health Improvement (DECIPHer), a UKCRC Public Health Research Centre of Excellence. Joint funding (MR/KO232331/1) from the British Heart Foundation, Cancer Research UK, Economic and Social Research Council, Medical Research Council, the Welsh Government and the Wellcome Trust, under the auspices of the UK Clinical Research Collaboration, is gratefully acknowledged.

.

DATA SHARING STATEMENT

No additional data available.

COMPETING INTERESTS

The authors declare that they have no competing interests.

KEY MESSAGES

What is already known on this subject

- Many countries worldwide have introduced legislation banning smoking in public places.
- Short-term evaluations show that legislation was followed by small declines in childhood exposure to secondhand smoke.
- In many countries, increases in voluntary restrictions on smoking in cars and homes were observed after legislation.
- Many children, particularly from poorer backgrounds, continued to be exposed to SHS in cars and homes.

What important gaps in knowledge exist on this topic

For peer review only

- Little data is available on whether the de-normalisation of smoking in front of children observed after smoke-free legislation continued in the longer term.

What this study adds

- Seven years after implementation of smoke-free legislation in Wales, children's exposure to smoke in cars has halved, while smoking in the home has declined substantially.
- However, among children with parents who smoke, 1 in 5 continue to allow smoking in their car, while almost half continue to smoke in the home.
- Although declining across the socioeconomic spectrum, children from poorer families remain most likely to be exposed to secondhand smoke in their car or home.

REFERENCES

1. Department of Health and Committee on the Medical effects of Air Pollutants. Handbook on air pollution and health. London: The Stationery Office, 1997.

2. Scientific Committee on Tobacco and Health. Update of evidence of health effects of secondhand smoke. London: Department of Health, 2004.

3. World Health Organisation. WHO Framework Convention on Tobacco Control. Geneva: WHO Document Production Services, 2005.

4. Hyland A, Barnoya J, Corral JE. Smoke-free air policies: past, present and future. Tobacco Control 2012;**21**(2):154-61.

5. Öberg M, Jaakkola MS, Woodward A, et al. Worldwide burden of disease from exposure to second-hand smoke: a retrospective analysis of data from 192 countries. The Lancet 2011;**377**(9760):139-46.

6. Ho SY, Wang MP, Lo WS, et al. Comprehensive smoke-free legislation and displacement of smoking into the homes of young children in Hong Kong. Tobacco Control 2010;**19**(2):129-33.

For peer review only

7. Adda J, Cornaglia FJ. The Effect of Taxes and Bans on Passive Smoking. IZA Discussion Paper No. 2191. . Secondary The Effect of Taxes and Bans on Passive Smoking. IZA Discussion Paper No. 2191. 2006. Available at SSRN: http://ssrn.com/abstract=919963.

8. Akhtar PC, Haw SJ, Currie DB, et al. Smoking restrictions in the home and secondhand smoke exposure among primary schoolchildren before and after introduction of the Scottish smoke-free legislation. Tobacco control 2009;**18**(5):409-15.

9. Phillips R, Amos A, Ritchie D, et al. Smoking in the home after the smoke-free legislation in Scotland: qualitative study. bmj 2007;**335**(7619):553.

10. Jarvis MJ, Sims M, Gilmore A, et al. Impact of smoke-free legislation on children's exposure to secondhand smoke: cotinine data from the Health Survey for England. Tobacco Control 2011.

11. Moore GF, Currie D, Gilmore G, et al. Socioeconomic inequalities in childhood exposure to secondhand smoke before and after smoke-free legislation in three UK countries. Journal of Public Health 2012;**34**(4):599-608.

12. Moore GF, Holliday JC, Moore LAR. Socioeconomic patterning in changes in child exposure to secondhand smoke after implementation of smoke-free legislation in Wales. Nicotine & Tobacco Research 2011;**13**(10):903-10.

13. Mons U, Nagelhout GE, Allwright S, et al. Impact of national smoke-free legislation on home smoking bans: findings from the International Tobacco Control Policy Evaluation Project Europe Surveys. Tobacco Control 2013;**22**(e1):e2-e9.

14. Cheng K-W, Glantz SA, Lightwood JM. Association between smokefree laws and voluntary smokefree-home rules. American journal of preventive medicine 2011;**41**(6):566-72.

For peer review only

15. Akhtar PC, Currie DB, Currie CE, et al. Changes in child exposure to environmental tobacco smoke (CHETS) study after implementation of smoke-free legislation in Scotland: national cross sectional survey. BMJ 2007;**335**(7619):545.

16. Holliday J, Moore G, Moore L. Changes in child exposure to secondhand smoke after implementation of smoke-free legislation in Wales: a repeated cross-sectional study. BMC Public Health 2009;**9**(1):430.

17. Freeman B, Chapman S, Storey P. Banning smoking in cars carrying children: an analytical history of a public health advocacy campaign. Australian and New Zealand journal of public health 2008;**32**(1):60-65.

18. Semple S, Apsley A, Galea KS, et al. Secondhand smoke in cars: assessing children's potential exposure during typical journey conditions. Tobacco Control 2012;**21**(6):578-83.

19. ASH Scotland. Secondhand smoke in cars. Edinburgh, 2011.

20. Hitchman SC, Fong GT, Zanna MP, et al. Support and correlates of support for banning smoking in cars with children: findings from the ITC Four Country Survey. The European Journal of Public Health 2011;**21**(3):360-65.

21. Roberts. C., Kawol. K. Smoking in Cars Carrying Children: monitoring public attitudes, November 2013 update, 2014.

22. Thomson G, Wilson N. Public attitudes to laws for smoke-free private vehicles: a brief review. Tobacco control 2009;**18**(4):256-61.

23. Leatherdale ST, Smith P, Ahmed R. Youth exposure to smoking in the home and in cars: how often does it happen and what do youth think about it? Tobacco control 2008;**17**(2):86-92.

24. British Medical Association Board of Science. Smoking in vehicles: A briefing from the Board of Science: British Medical Association,, 2011.

For peer review only

25. Hopkinson NS, Majeed A, Britton J, et al. Respiratory health professionals call on MPs to vote to ban smoking in cars with children. BMJ: British Medical Journal 2014;**348**.

26. Dolcini MM, Adler NE, Lee P, et al. An assessment of the validity of adolescent self-reported smoking using three biological indicators. Nicotine & Tobacco Research 2003;**5**(4):473-83.

27. Currie C, Molcho M, Boyce W, et al. Researching health inequalities in adolescents: The development of the Health Behaviour in School-Aged Children (HBSC) Family Affluence Scale. Social Science & Medicine 2008;**66**(6):1429-36.

For peer review only

STROBE Statement—checklist of items that should be included in reports of observational studies

	Item No	Recommendation
**Title and abstract**	1	(*a*) Indicate the study's design with a commonly used term in the title or the abstract. x
		(*b*) Provide in the abstract an informative and balanced summary of what was done and what was found. x
**Introduction**		
Background/rationale	2	Explain the scientific background and rationale for the investigation being reported. x
Objectives	3	State specific objectives, including any prespecified hypotheses. x
**Methods**		
Study design	4	Present key elements of study design early in the paper x
Setting	5	Describe the setting, locations, and relevant dates, including periods of recruitment, exposure, follow-up, and data collection x
Participants	6	(*a*) *Cohort study*—Give the eligibility criteria, and the sources and methods of selection of participants. Describe methods of follow-up<br>*Case-control study*—Give the eligibility criteria, and the sources and methods of case ascertainment and control selection. Give the rationale for the choice of cases and controls<br>*Cross-sectional study*—Give the eligibility criteria, and the sources and methods of selection of participants x
		(*b*) *Cohort study*—For matched studies, give matching criteria and number of exposed and unexposed<br>*Case-control study*—For matched studies, give matching criteria and the number of controls per case
Variables	7	Clearly define all outcomes, exposures, predictors, potential confounders, and effect modifiers. Give diagnostic criteria, if applicable x
Data sources/ measurement	8*	For each variable of interest, give sources of data and details of methods of assessment (measurement). Describe comparability of assessment methods if there is more than one group x
Bias	9	Describe any efforts to address potential sources of bias x
Study size	10	Explain how the study size was arrived at x
Quantitative variables	11	Explain how quantitative variables were handled in the analyses. If applicable, describe which groupings were chosen and why x
Statistical methods	12	(*a*) Describe all statistical methods, including those used to control for confounding x
		(*b*) Describe any methods used to examine subgroups and interactions x
		(*c*) Explain how missing data were addressed x
		(*d*) *Cohort study*—If applicable, explain how loss to follow-up was addressed<br>*Case-control study*—If applicable, explain how matching of cases and controls was addressed<br>*Cross-sectional study*—If applicable, describe analytical methods taking account of sampling strategy x
		(*e*) Describe any sensitivity analyses

Continued on next page

For peer review only - http://bmjopen.bmj.com/site/about/guidelines.xhtml

**Results**		
Participants	13*	(a) Report numbers of individuals at each stage of study—eg numbers potentially eligible, examined for eligibility, confirmed eligible, included in the study, completing follow-up, and analysed x
		(b) Give reasons for non-participation at each stage x
		(c) Consider use of a flow diagram n/a
Descriptive data	14*	(a) Give characteristics of study participants (eg demographic, clinical, social) and information on exposures and potential confounders x
		(b) Indicate number of participants with missing data for each variable of interest x
		(c) *Cohort study*—Summarise follow-up time (eg, average and total amount) n/a
Outcome data	15*	*Cohort study*—Report numbers of outcome events or summary measures over time
		Case-control study—Report numbers in each exposure category, or summary measures of exposure
		Cross-sectional study—Report numbers of outcome events or summary measures x
Main results	16	(*a*) Give unadjusted estimates and, if applicable, confounder-adjusted estimates and their precision (eg, 95% confidence interval). Make clear which confounders were adjusted for and why they were included x
		(*b*) Report category boundaries when continuous variables were categorized x
		(*c*) If relevant, consider translating estimates of relative risk into absolute risk for a meaningful time period n/a
Other analyses	17	Report other analyses done—eg analyses of subgroups and interactions, and sensitivity analyses x
**Discussion**		
Key results	18	Summarise key results with reference to study objectives x
Limitations	19	Discuss limitations of the study, taking into account sources of potential bias or imprecision. Discuss both direction and magnitude of any potential bias x
Interpretation	20	Give a cautious overall interpretation of results considering objectives, limitations, multiplicity of analyses, results from similar studies, and other relevant evidence x
Generalisability	21	Discuss the generalisability (external validity) of the study results x
**Other information**		
Funding	22	Give the source of funding and the role of the funders for the present study and, if applicable, for the original study on which the present article is based x

*Give information separately for cases and controls in case-control studies and, if applicable, for exposed and unexposed groups in cohort and cross-sectional studies.

Note: An Explanation and Elaboration article discusses each checklist item and gives methodological background and published examples of transparent reporting. The STROBE checklist is best used in conjunction with this article (freely available on the Web sites of PLoS Medicine at http://www.plosmedicine.org/, Annals of Internal Medicine at http://www.annals.org/, and Epidemiology at http://www.epidem.com/). Information on the STROBE Initiative is available at www.strobe-statement.org.

For peer review only

BMJ Open

Prevalence of smoking restrictions and child exposure to secondhand smoke in cars and homes: a repeated cross-sectional survey of 10-11 year old children in Wales

Journal:	*BMJ Open*
Manuscript ID:	bmjopen-2014-006914.R1
Article Type:	Research
Date Submitted by the Author:	12-Dec-2014
Complete List of Authors:	Moore, Graham; Cardiff University, School of Social Sciences<br>Moore, L; University of Glasgow, MRC/CSO Social and Public Health Sciences Unit<br>Littlecott, Hannah; Cardiff University, School of Social Sciences<br>Ahmed, Nilufar; Cardiff University, Social Sciences<br>Lewis, Sophia; Cardiff University, School of Social Sciences<br>Sulley, Gillian<br>Jones, Elen<br>Holliday, Jo; Cardiff University, Social Sciences
**Primary Subject Heading**:	Public health
Secondary Subject Heading:	Smoking and tobacco
Keywords:	PUBLIC HEALTH, PREVENTIVE MEDICINE, SECONDHAND SMOKE

SCHOLARONE™
Manuscripts

Prevalence of smoking restrictions and child exposure to secondhand smoke in cars and homes: a repeated cross-sectional survey of 10-11 year old children in Wales

CORRESPONDING AUTHOR

Graham F Moore

Address: Centre for the Development and Evaluation of Complex Interventions for Public Health Improvement (DECIPHer), 1-3 Museum Place, Cardiff University, School of Social Sciences, Cardiff, CF10 3BD

Email: MooreG@cf.ac.uk

Telephone number: 02920 875360

CO-AUTHORS

Laurence Moore

MRC/CSO Social & Public Health Sciences Unit, University of Glasgow, UK

Hannah J Littlecott

Centre for the Development and Evaluation of Complex Interventions for Public Health Improvement (DECIPHer), Cardiff University, UK

Nilufar Ahmed

Centre for the Development and Evaluation of Complex Interventions for Public Health Improvement (DECIPHer), Cardiff University, UK

Sophia Lewis

School of Social Sciences, Cardiff University, UK

Gillian Sulley

Centre for the Development and Evaluation of Complex Interventions for Public Health Improvement (DECIPHer), Cardiff University, UK

Elen Jones

Centre for the Development and Evaluation of Complex Interventions for Public Health Improvement (DECIPHer), Cardiff University, UK

Jo Holliday

Centre for the Development and Evaluation of Complex Interventions for Public Health Improvement (DECIPHer), Cardiff University, UK

KEYWORDS

For peer review only

Secondhand smoke, public policy, prevention, children.

WORD COUNT

3960

For peer review only

ABSTRACT

Objective

Small increases in smoking restrictions in cars and homes were reported after legislation prohibiting smoking in public places. Few studies examine whether these changes continued in the longer term. This study examines changes in restrictions on smoking in cars and homes, and child exposure to SHS in these locations, since 2008 post-legislation surveys in Wales.

Setting

State-maintained primary schools in Wales (n=75).

Participants

Children aged 10-11 years (Year 6) completed CHETS (CHild exposure to Environmental Tobacco Smoke) Wales surveys in 2007 (n=1612) and 2008 (n=1605). A replication survey (CHETS Wales 2) was conducted in 2014, including 1601 children.

Primary outcome variable

Children's reports of whether smoking was allowed in their car or home and exposure to SHS in a car or home the previous day.

Results

The percentage of children who reported that smoking was allowed in their family vehicle fell from 18% to 9% in 2014 (OR=0.42; 95% CI=0.33 to 0.54). The percentage living in homes where smoking was allowed decreased from 37% to 26% (OR=0.30; 95% CI=0.20 to 0.43). Among children with a parent who smoked, 1 in 5 and 1 in 2 continued to report that smoking was allowed in their car and home. The percentage reporting SHS exposure in a car (OR=0.52; 95% CI=0.38 to 0.72) or home (OR=0.44; 95% CI=0.36 to 0.53) the previous day also fell. Children from poorer families remained less likely to report smoking restrictions.

Conclusions

For peer review only

Smoking in cars and homes has continued to decline. Substantial numbers of children continue to report that smoking is allowed in cars and homes, particularly children from poorer families. A growing number of countries have legislated, or plan to legislate, banning smoking in cars carrying children. Attention is needed to the impact of legislation on child health and health inequalities, and reducing smoking in homes.

STRENGTHS AND LIMITATIONS

- The study reports findings from a survey of a large (n=1601) nationally representative sample of 10-11 year old children in Wales, replicating earlier surveys in 2007/8.
- Repeated cross sectional surveys were conducted with the same schools in 2007/08. More than two-thirds of those same schools were recruited in 2014. Remaining schools were replaced by schools from the same area and with comparable socioeconomic status. Samples were comparable on socio-demographic measures.
- The substantial differences in childhood reports of restrictions on smoking in cars and homes, and reports of exposure to SHS in a car or home the previous day, between 2008 and 2014 surveys can therefore confidently be said to represent change over time.
- The study is limited by reliance on self-report measures of smoking restrictions and SHS exposure, though measures are validated against cotinine data collected in 2007/8.
- It is not possible to make causal attributions regarding how changes over time came about.

For peer review only

BACKGROUND

The dangers of secondhand smoke (SHS, or passive smoking) are now well established.[1 2] Indeed, the World Health Organisation (WHO) state that that 'scientific evidence has unequivocally established that exposure to tobacco smoke causes death, disease and disability'.[3] Growing recognition of the dangers of SHS led many countries, including all UK countries, to implement legislation prohibiting smoking in enclosed public places and workplaces in the last decade; by 2011, an estimated 11% of the world's population lived in countries where smoking was prohibited in public spaces.[4]

In 2004, it was estimated that 61% of disease caused by SHS exposure worldwide was borne by children,[5] whose developing lungs and rapid breathing rate make them particularly vulnerable to SHS.[6] Hence, while smoke-free legislation was implemented with the primary objective of protecting adults such as hospitality workers, impacts on childhood SHS received significant international scrutiny. The case against legislation made by its opponents centred on arguments that banning smoking in public spaces would displace smoking into the home. Some evidence to support this claim was reported in Hong Kong[7] and the USA.[8] However, studies in all UK countries contradicted the displacement hypothesis. Increases in the adoption of voluntary home smoking restrictions were reported in Scotland[9 10] and England.[11] While in Wales the proportion of homes with full smoking restrictions did not change significantly,[12] fewer children reported that parents smoked inside the home after legislation.[13] Indeed, a growing body of international evidence indicates that smoke-free legislation was, in most cases, followed by increases in voluntary restrictions on smoking in private spaces.[14 15]

For peer review only

While the growing de-normalisation of smoking around children reflected by these trends is welcome, declines in childhood SHS exposure immediately after legislation primarily benefited groups who were at relatively low risk prior to legislation. Significant declines occurred primarily among children of non-smokers[16 17] and from more affluent families.[12 13] Substantial percentages of children continued to report exposure to SHS in homes and cars. In Wales for example, 1 in 5 children reported that smoking was allowed in their family car, while more than a third reported living in homes where smoking was allowed.[12] All measures of restrictions on smoking and childhood exposure to SHS in homes and cars indicated that, before and after legislation, exposure was particularly prevalent among children from poorer families.[12]

Debates regarding how to safeguard children from the dangers of SHS, and address the role of SHS in the intergenerational reproduction of socioeconomic inequalities, have therefore moved toward attempts to reduce smoking in cars and homes. Due to the private nature of these spaces, regulation of behaviour is often regarded as an invasion of privacy. Hence, legislation will often only be considered where efforts to achieve change via voluntary means have not fully addressed the problem. In particular, while homes remain children's main source of SHS exposure, some have argued that only the in the most authoritarian of states would legislation around smoking in the home be acceptable.[18] Hence, efforts to promote smoke-free homes remain focused on voluntary rather than legislative means.[19]

However, cars represent a space in which behaviours are already heavily regulated, hence occupying an intermediate space between public and private.[18] While children are likely to spend less time exposed to SHS inside cars than inside homes, the small and enclosed nature of vehicles means that SHS exposure is likely to be of an intense nature.[20] Furthermore, there

For peer review only

is tentative evidence of spill-over effects of banning smoking in cars, with one survey from the US showing a substantial increase in adoption of home smoking restrictions after state-wide legislation on smoking in vehicles.[21] Hence, in a growing number of countries including parts of Australia, Canada and the USA,[22] bans have been introduced on smoking in cars carrying children. Recent surveys indicate widespread public support for such a ban,[23, 24] while organisations including the British Medical Association have called for a ban on smoking in all vehicles.[25] More recently, a call was issued by 600 UK respiratory health professionals for MPs to back a ban on smoking in cars carrying children.[26]

In England, a House of Commons vote in 2014 gave ministers the power to introduce a ban on smoking in cars carrying children. In Wales, the Welsh Government have attempted to restrict smoking in cars via voluntary means, announcing plans for the 'Fresh Start Wales' campaign in October 2011. This campaign, launched in 2012, comprised a range of marketing techniques through multimedia advertisements with the tagline 'Smoking in your car poisons your children', signposting to services that support quitting. The Welsh Government indicated that if insufficient voluntary changes were observed over the following 3 years, legislation would be considered, with the Children and Families Act of 2014 giving Welsh Ministers the authority to pass such legislation.

This paper presents findings of a replication of the earlier CHETS Wales surveys commissioned by the Welsh Government to assist with informing a decision on whether to proceed with legislation. It examines changes in children's reports of smoking restrictions and exposure to smoke in cars and homes, whether socioeconomic patterning in these variables has changed over time, and children's own attitudes towards a possible ban on smoking in cars. In summary, the paper addresses the following key research questions:

For peer review only

- Have the adoption of smoking restrictions in cars and homes increased (and children's reported exposure to SHS in these locations decreases) in Wales from 2008 to 2014?
- Have socioeconomic inequalities narrowed, widened or remained the same?
- Are increases in smoking restrictions in private spaces reported by children with parents who smoke?
- What are children's views on whether or not smoking in cars should be banned?

METHODS

Study design

CHETS Wales was a repeated cross-sectional study of Year 6 (age 10-11 years) schoolchildren in 2007 and 2008. A replication study (CHETS Wales 2) was commissioned to assess changes in smoking in cars and other private spaces in 2014. Both were reviewed and approved by the Cardiff University School of Social Sciences Research Ethics Committee.

Sampling

CHETS Wales recruited a nationally representative sample of 75 state maintained primary schools across Wales. Schools were stratified according to high/low (cut off point identified as average entitlement across whole sample; 17.12%) free school meal entitlement (as a proxy for socioeconomic status) and Local Education Authority. Within each stratum, schools were selected on a probability proportional to school size. Where schools declined to participate, replacement schools were identified from within the same stratum. For CHETS Wales, target sample sizes were based on power to detect change in overall SHS exposure, assessed salivary cotinine. While CHETS Wales 2 was focused on reported SHS exposure in

For peer review only

specific locations, hence using questionnaire data, it replicated the sampling methods used for CHETS Wales. The same schools who took part in CHETS Wales were approached where possible. Schools who declined or could not be contacted were replaced with another school sampled from the same stratum. Schools were paid £50 each for their time. Within each school, one Year 6 (age 10-11) class was randomly selected to participate, with all students in the class being involved.

Consent and data collection

Consent and data collection procedures for CHETS Wales are described in detail elsewhere.[17] These were replicated for CHETS Wales 2, with the exception that no saliva samples were collected. In brief, consent was sought from schools and parents, and assent from children. Schools signed a written agreement. An opt-out consent procedure was used for parental consent in the majority of schools, with a small number requesting use of opt-in consent. Children were also assured that their participation was voluntary and given the opportunity to opt-out on the day. In all years, data were collected over a ten week period between February to April in each year of collection. Data were collected in the classroom environment by trained staff. All staff were provided with a data collection protocol and given training in the DECIPHer centre to maximise standardisation of data collection procedures across the schools and data collection sweeps. Class teachers were asked to be present for disciplinary purposes, but not to intervene in the data collection in any other way unless asked to do so by the member of the research team.

Variables

Smoking in cars and the home

Children were asked ‘Is smoking allowed in your family car, van or truck? (‘yes’, ‘no’, ‘I don’t know’ or ‘don’t have a family car, van or truck’) as well as ‘While you were inside a

For peer review only

car yesterday was anyone smoking there?'. Home smoking restrictions were assessed by asking children 'Is smoking allowed inside your home?' ('No, smoking is not allowed at all', 'smoking is allowed in certain areas only', 'smoking is allowed anywhere in our home', 'smoking is allowed only on special occasions in our home', 'I don't know'). Children were also asked 'While you were inside your home yesterday was anyone smoking there?'. Parental smoking in the home was assessed with the question 'Do any of the following people smoke in the home?' in relation to i) father, ii) mother, iii) stepfather (or mother's partner), iv) stepmother (or father's partner) with response options 'smokes every day', 'smokes sometimes', 'does not smoke', 'I don't know', 'I don't have or see this person'. The parent was classified as smoking in the home if the child responded 'smokes every day' or 'smokes sometimes'. Children were categorized as having i) no parent figures who smoke in the home, ii) a father figure only who smokes in the home, iii) a mother figure only who smokes in the home, iv) two parent figures who smoke in the home.

Objectively measured secondhand smoke exposure

Salivary cotinine (a metabolite of nicotine) is a well-validated biomarker of SHS exposure in the previous 72 hours [27]. Anonymous samples were assayed using capillary gas chromatography with a detection limit of 0.1ng/ml. Saliva samples were collected in 2007 and 2008, but not 2014. Hence, they are used to indicate the validity of self-reports of smoking in cars and homes.

Attitudes to banning smoking in cars

In 2014, children's attitude to banning smoking in cars were assessed by asking children to circle (on a scale of 1-5) how much they agreed or disagreed with the following statements: 'There should be a complete ban on smoking in cars'; 'Smoking should be banned in cars carrying children under 16'.

Child smoking behaviour

For peer review only

Respondent smoking behaviour was measured using the Office for National Statistics scale.[28] Students who gave a response other than 'I do not smoke' were classified as smokers. Additional options were 'every day', 'at least once a week', or 'less than once a week'.

Socioeconomic status

Children completed the Family Affluence Scale (FAS[29]), which generates a composite scale based on responses to questions on bedroom occupancy, car and computer ownership, and holidays. Items were summed to form a total FAS score.

Age

Children were asked to indicate the year and month of their birth on the smoking questionnaire. The month that the questionnaire was completed was recorded, and children's age in years calculated.

Statistical analysis

Descriptive statistics are presented to examine the comparability of samples at 2007, 2008 and 2014 in terms of sex, age, socioeconomic status, family structure and child smoking status. Significance of difference between survey years is tested using design-adjusted chi-squared analyses for categorical variables and t-tests for age. For all key variables other than parental smoking in the home (6.0%), data were missing in less than 5% of cases. The validity of self-report items used to assess smoking in cars and homes was examined by presenting median and interquartile range cotinine values, as well as the percentage of children whose saliva samples contained detectable traces of cotinine, by reported exposure. Subsequently, frequencies and percentages of children who reported exposure to secondhand smoke in cars and homes were calculated for all three time-points. Significance of change from 2008 to 2014 was evaluated using logistic regression models adjusted for age and family affluence, with the year of data collection entered as the primary independent variable.

For peer review only

Odds ratios represent the odds of a child reporting exposure to SHS in the location specified in 2014 relative to 2008. To account for the clustered nature of the data sample, random terms for school were included in all models. These analyses were run twice: firstly with the entire sample, and secondly limited to children with at least one smoking parent. The above models were also used to examine socioeconomic inequality in smoke exposure in private spaces, through inclusion of FAS scores in the models, and testing of FAS by survey year interactions. For consistency with earlier analyses of CHETS Wales data, models including family affluence terms were limited to children living with one or both parent figures, although sensitivity analyses indicated that models which did or did not exclude children in other living arrangements gave consistent results. As a further sensitivity analysis, regression models examining change from 2008 to 2014 were re-run using only the 51 schools who took part in both years. As these produced comparable results, we report only the models using the full sample.

RESULTS

Response rates

Response rates for CHETS Wales are reported in detail elsewhere. In brief, 75 of 119 schools approached participated (63.0%) at both time-points, with child level response rates of 91.5% and 90.4% respectively. Of the 75 schools who participated in CHETS Wales, four could not be invited to participate in CHETS Wales 2 due to closure or change in status (i.e. no longer a mainstream school). Of the remaining schools, 51 participated. Forty-three further schools were invited to participate before the target of 75 schools was reached (overall response rate=65.8%). Of 1862 pupils within selected classes, completed questionnaires were obtained from 1601 (86.0%). In schools where opt-out consent procedures were followed (n=74 schools, 1810 pupils), 56 children were opted-out by parents, 35 children refused, and 141

For peer review only

were absent on the day of collection. Data were obtained from 1578 pupils (87.2%). One school requested opt in consent. Of the 52 eligible pupils in this school, consent was given for 23 children (44.2%), all of whom provided data.

Sample description

Pupil demographics at each time-point are presented in Table 1. There were no significant differences between time-points, with the exception of FAS scores, which were highest in 2014. However, this was explained entirely by widespread computer ownership in 2014, with FAS scores almost identical at all time-points where this item was removed. Hence, for analyses using FAS, this item is removed. FAS scores with or without computers were highly correlated (r=0.87). There were also no significant demographic differences between children within schools that participated at all time-points and children within schools which did not participate again in 2014 (compared using 2008 data) or replacement schools (compared using 2014 data).

For peer review only

Table 1. Sample descriptions by survey year. Figures are frequencies (and percentages) unless otherwise indicated

	Survey year			P-values for tests of difference		
	**2007 (n=1612)**	**2008 (n=1605)**	**2014 (n=1601)**	**Comparison between years**	**Schools who did vs did not participate in 2014 (2008 data)**	**Original vs replacement schools (2014 data)**
Boys	778 (48.5)	792 (49.4)	797 (49.8)	0.80	0.53	0.75
Mean (SD) age	10.9 (0.4)	10.9 (0.4)	10.9 (0.4)	0.42	0.71	0.54
Mean (SD) FAS score	5.6 (1.9)	5.7 (1.9)	6.6 (1.9)	<0.001	0.43	0.90
Mean (SD) FAS score without computers	3.9 (1.5)	3.9 (1.4)	3.9 (1.4)	0.41	0.93	0.71
Two parent families	1120 (69.5)	1089 (67.9)	1074 (67.1)	0.37	0.12	0.57
Step families	170 (10.6)	175 (10.9)	152 (9.4)			
Single mother	263 (16.3)	273 (17.0)	282 (17.6)			
Single father	18 (1.1)	23 (1.4)	32 (2.0)			
Self-reported smokers	24 (1.5)	18 (1.1)	12 (0.8)	0.19	0.28	0.30

p-values for design adjusted chi-squared analyses, except for age (t-test)

For peer review only

Validity of self-reported measures of smoking restrictions and SHS exposure

Median and interquartile range salivary cotinine values (using 2007-08 data), broken down by responses to self-report measures of smoking restrictions and SHS exposure are presented in Table 2. In addition, percentages of children with cotinine above the limit of detection are presented. In all cases, children who reported no smoking restrictions, or being exposed to SHS in homes or cars, provided samples with higher cotinine concentrations and were substantially more likely to provide samples containing a detectable level of cotinine. Where limited to children who reported that smoking was allowed in their home, median cotinine concentrations were 7 times higher where children reported that smoking was also allowed in their car by comparison to those who said it was not (1.3ng/ml vs 0.2ng/ml), and twice as high for children who reported being in a car where someone was smoking the previous day versus those who did not (1.6ng/ml vs 0.8ng/ml). Hence, items on smoking in cars reflected differences in objectively measured SHS exposure which were not explained by the fact that most children who reported exposure to SHS in cars were also exposed to SHS in the home.

For peer review only

Table 2. Salivary cotinine concentrations by responses to self-report items on exposure to SHS in cars and homes

		Median (and inter-quartile range) salivary cotinine concentration (ng/ml)	Frequency and percentage cotinine above Limit of Detection	P-value
Smoking allowed in car	No (n=1689)	<0.1 (<0.1 to 0.2)	594 (35.2)	
	Yes (n=569)	1.1 (0.4 to 2.2)	526 (92.4)	<0.001
	Don't know (n=424)	0.1 (<0.1 to 0.8)	235 (55.4)	
	Don't own a car (n=211)	1.1 (0.2 to 2.7)	179 (84.8)	
In a car where someone was smoking yesterday	No (n=2653)	<0.1 (<0.1 to 0.6)	1320 (49.8)	
	Yes (n=196)	1.4 (0.7 to 2.9)	186 (94.9)	<0.001
Parent figures smoke in the home	None (n=1781)	<0.1 (<0.1 to 0.1)	588 (33.0)	
	Father (n=272)	0.5 (0.1 to 1.2)	225 (82.7)	
	Mother (n=299)	1.2 (0.4 to 2.2)	274 (91.6)	<0.001
	Both (n=406)	1.8 (1.0 to 3.0)	396 (96.3)	
Smoking restrictions in the home	Full (n=1557)	<0.1 (<0.1 to 0.1)	484 (31.1)	
	Partial (n=672)	0.5 (0.1 to 1.6)	534 (79.5)	<0.001
	None (n=337)	1.7 (0.9 to 2.9)	319 (94.7)	

*p-values from design-adjusted chi-squared analyses

Changes in smoking restrictions and self-reported exposure to SHS in cars and homes

Table 3 indicates that restrictions on smoking in cars have increased substantially since 2008, with small increases between 2007 and 2008, and more rapid changes since. For example, in 2014, 9% of children (11% of those who reported that their family own a vehicle and that they know whether or not smoking is allowed in it) reported that smoking was allowed in it, a decline from 18% (23%) in 2008. Similar declines were observed among children of smokers, though 1 in 5 continued to report that smoking was allowed in their family vehicle. In 2014, 4% of all children, and 7% of children of smokers reported having been in a car where someone was smoking the previous day; a halving of exposure since 2008.

For peer review only

Table 3. Frequency (and percentage) of 10-11 year old children in Wales reporting smoking restrictions in car

		Smoking allowed in family car?				In car where someone smoking yesterday?
		Yes	No	Don't know	No car	
Whole sample	2007	327 (20.4)	926 (57.8)	231 (14.4)	118 (7.4)	107 (6.9)
	2008	288 (18.0)	965 (60.3)	234 (14.6)	114 (7.1)	107 (6.7)
	2014	141 (8.9)	1140 (71.7)	195 (12.3)	115 (7.2)	57 (3.6)
P-value*		<0.001				<0.001
Children with a parent who smokes	2007	301 (38.6)	272 (34.9)	114 (14.6)	92 (11.8)	102 (13.5)
	2008	259 (34.8)	284 (38.2)	123 (16.5)	78 (10.4)	98 (13.3)
	2014	131 (19.6)	371 (55.5)	87 (13.0)	79 (11.8)	46 (7.0)
P-value*		P<0.001				P<0.001

*p-values from design-adjusted chi-squared analyses

As indicated in Table 4, percentages of children living in 'smoke-free' homes (i.e. homes where smoking is not allowed at all) increased slightly between 2007 and 2008, though more markedly between 2008 and 2014. Similar changes were observed for children of smokers, among whom, half reported living in a smoke free home in 2014, compared to 1 in 3 in 2008, while 1 in 11 lived in a home with no smoking restrictions, compared to 1 in 4 in 2008. Table 4 also indicates small declines in percentages of children reporting that one or more parent figures smoked, falling from 47% in 2007 to 40% in 2014. Larger declines were observed in percentages reporting that one or more parent figures smoked in the home. Figures for children with a parent who smoked indicate substantial reductions in the proportion of children of smokers whose parents smoked in the home, falling from 74% in 2007 to 71% in 2008 and to 52% in 2014. Hence, by 2014, almost half of children who reported that at least one parent figure smoked, reported that those parent figures did not smoke in the home. The percentage of children reporting that someone was smoking in their home the previous day

For peer review only

while they were present fell only slightly from 20.7% (n=328) in 2007, to 19.8% (n=313) in 2008 and halved to 9.6% (n=148) in 2014.

Table 4. Frequency (and percentage) of 10-11 year old children in Wales reporting that parent figures smoke and levels of smoking restrictions in the home

	No smoking parent figure	Father smokes	Mother smokes	Both smoke	P-value
2007	825 (52.8)	230 (14.7)	187 (12.0)	322 (20.6)	0.01
2008	858 (55.5)	235 (15.2)	187 (12.1)	267 (17.3)	
2014	929 (60.2)	211 (13.7)	164 (10.6)	240 (15.5)	
	No parent figure smokes in home	Father smokes in home	Mother smokes in home	Both smoke in home	
All children					
2007	973 (63.2)	148 (9.6)	161 (10.5)	258 (16.8)	<0.001
2008	1009 (66.8)	144 (9.5)	164 (10.9)	194 (12.8)	
2014	1153 (78.0)	93 (6.3)	91 (6.2)	141 (9.5)	
Children with one or more parents who smoke					
2007	192 (25.7)	142 (19.0)	158 (21.2)	254 (34.1)	<0.001
2008	201 (29.2)	138 (20.1)	159 (23.1)	190 (27.6)	
2014	289 (47.7)	92 (15.2)	88 (14.5)	137 (22.6)	
	Smoking in the home				
	Full restriction	Partial restriction	No restriction		
All children					
2007	841 (59.1)	385 (27.1)	196 (13.8)		<0.001
2008	883 (62.7)	361 (25.6)	164 (11.7)		
2014	1041 (74.3)	303 (21.6)	57 (4.1)		
Children with one or more parents who smoke					
2007	220 (32.0)	285 (41.5)	182 (26.5)		<0.001
2008	218 (33.7)	278 (43.0)	151 (23.3)		
2014	294 (51.0)	231 (40.0)	52 (9.0)		

*p-values from design-adjusted chi-squared analyses

Table 5 presents odds ratios and 95% confidence intervals from logistic regression models, examining change over time from 2008-2014 in the variables described in Tables 3 and 4, and associations of socioeconomic status (FAS score) with smoking in private spaces. These analyses show that all markers of exposure to SHS in cars and homes decreased significantly from 2008 to 2014. These results were maintained when the sample was restricted to those

For peer review only

children with at least one parent figure who smokes. The likelihood of a child reporting exposure to SHS was significantly lower for children from more affluent families in relation to all measures of exposure. There were no significant interactions between SES and survey year, with the exception of the percentage of children reporting being in a car the previous day where someone was smoking, for which socioeconomic inequalities narrowed significantly. For all remaining measures of SHS exposure, there were no significant reductions or increases in inequality.

Children's views on smoking in cars in 2014

Among the whole sample, 71.2% (n=1109) of children agreed that smoking should be banned in cars, with 76.4% (n=1191) agreeing that smoking should be banned in cars if children were present. Where limited to children who reported that smoking was allowed in their family vehicle, a small majority agreed that smoking should be banned in all cars (55.4%; n=77) while a larger majority (61.9%; n=86) agreed that smoking should be banned in cars when children are present.

For peer review only

Table 5. Odds ratios and 95% confidence intervals from logistic regression models examining associations of year of data collection and SES with exposure to smoke in private spaces

		Smoking allowed in cars (yes vs no)	Smoking in car yesterday	Smoking restriction in the home (base category=full restriction)		Smoking in home yesterday	Parent figures smoke in the home		
				Partial	No restriction		Father only	Mother only	Both parents
	All children								
	n	**2407**	**2987**	**2664**	**2664**	**2955**	**2836**	**2836**	**2836**
Model 1	Year (ref=2008)	**0.42 (0.33 to 0.54)**	**0.52 (0.38 to 0.72)**	**0.70 (0.59 to 0.83)**	**0.30 (0.20 to 0.43)**	**0.44 (0.36 to 0.53)**	**0.54 (0.42 to 0.70)**	**0.48 (0.36 to 0.64)**	**0.65 (0.49 to 0.86)**
	FAS	**0.74 (0.68 to 0.80)**	0.92 (0.83 to 1.02)	**0.77 (0.72 to 0.83)**	**0.63 (0.57 to 0.71)**	**0.70 (0.65 to 0.75)**	**0.73 (0.67 to 0.81)**	**0.72 (0.65 to 0.79)**	**0.67 (0.62 to 0.73)**
Model 2	FAS*Year	1.14 (0.95 to 1.37)	**1.28 (1.01 to 1.60)**	1.00 (0.88 to 1.14)	1.05 (0.80 to 1.38)	1.06 (0.91 to 1.22)	0.94 (0.78 to 1.15)	1.06 (0.84 to 1.34)	1.05 (0.86 to 1.29)
	Children with at least one parent figure who smokes								
	n	**982**	**1303**	**1149**	**1149**	**1303**	**1217**	**1217**	**1217**
	Year (ref=2008)	**0.41 (0.31 to 0.53)**	**0.49 (0.35 to 0.69)**	**0.59 (0.48 to 0.73)**	**0.26 (0.17 to 0.39)**	**0.41 (0.33 to 0.51)**	**0.45 (0.33 to 0.60)**	**0.38 (0.28 to 0.53)**	**0.52 (0.38 to 0.70)**
	FAS	**0.87 (0.79 to 0.97)**	1.07 (0.96 to 1.20)	**0.82 (0.75 to 0.88)**	**0.70 (0.61 to 0.80)**	**0.80 (0.75 to 0.87)**	**0.88 (0.78 to 0.98)**	**0.86 (0.77 to 0.95)**	**0.79 (0.71 to 0.88)**

All models adjusted for age and include random terms for school. Significant ORs highlighted in bold

For peer review only

DISCUSSION

The findings presented in this paper suggest that the de-normalisation of smoking in enclosed spaces where children are present observed immediately after introduction of smoke-free legislation has continued.[11] The proportion of children who report that smoking is allowed in their family car has halved, while the percentage of children living in smoke-free homes has increased from less than 2 in 3 to almost 3 in 4. While in 2008 a clear majority of children who lived with a parent who smoked reported that smoking was allowed in their home,[12] half now report that their home is smoke free. While it is not possible to make firm causal attributions, it is possible that this represents a continuation of the effects of smoke-free legislation, and that evaluations included follow-up periods which were too short in duration to fully capture impacts. Notably however, other countries have reported more limited long-term progress in reducing smoking in cars and homes following smoke-free legislation; in New Zealand for example 23% of youth reported exposure to SHS in a car in the past week in 2012.[30]

While these trends are encouraging, a large proportion of children with a parent who smokes continue to report that smoking is allowed in their home (almost half) or family car (one in five). In light of the established harms of SHS,[1 2] these levels of smoking in cars and homes still represent a significant public health concern. Furthermore, consistent with aforementioned evidence from New Zealand,[30] adoption of smoke free homes continues to be significantly less common among children from poorer families. One recent paper argues that children from lower SES families are more likely to be exposed to SHS in part due to higher rates of parental smoking, but also that less affluent parents who smoke in their homes do so in greater proximity to their children, due to the smaller size of their homes.[31] Reducing

socioeconomic inequalities in children's exposure to tobacco, and to SHS, remain priorities in efforts to interrupt the intergenerational reproduction of inequality.

While efforts to promote smoking restrictions in the home continue to do so through promoting voluntary change, there is widespread support for a ban on smoking in cars, from health professionals and the public.[23-26 32] This study indicates support for such a ban from children themselves, with a large majority indicating that smoking in cars carrying children should not be allowed. Indeed, while fewer children who reported that smoking was allowed in their family car agreed with proposed legislation, a clear majority felt that smoking in cars carrying children should be banned.

Strengths of this study include its large nationally representative sample. While not all schools who took part in 2008 could be recruited again in 2014, the 2014 survey successfully recruited two-thirds of the schools who took part in the earlier CHETS Wales study, and achieved a sample with no significant demographic differences to the original sample. While we are unable to make causal attributions regarding how changes occurred, differences between survey years can be confidently considered to reflect change over time rather than sampling differences. The study relies upon self-reports of SHS exposure. However, while no saliva samples were collected in 2014, for all self-reported indicators of smoking restrictions and SHS exposure in cars and homes, objective indicators were consistent with children's reports in 2007/08. Hence, changes in self-reports of smoking restrictions and SHS exposure can be confidently assumed to reflect meaningful reductions in SHS exposure.

Partly informed by the key findings from this study, the Welsh Government announced that it will introduce legislation banning smoking in cars carrying children similar to that in place in

For peer review only

parts of Canada, Australia and the USA,[22] citing the high proportion of children with parents who smoke who are still exposed to smoke in cars. Further research is needed to understand the impacts of this legislation on childhood SHS exposure (including compliance with legislation, and effects on smoking behaviour in other locations, such as the home),[21] health outcomes and health inequalities. In addition, there is a need for sustained attention to understanding how to reduce smoking in the main location in which children continue to be exposed the SHS; the home. Further reducing childhood SHS exposure, while eliminating socioeconomic inequality, will likely require a combination of efforts to help parents to successfully quit smoking, and to support those who continue to smoke in not doing so in the home.

ACKNOWLEDGEMENTS

We thank Chris Roberts and Ian Jones (Social Research and Information Division, Welsh Government) for support and advice; and the Public Health Division (Welsh Government) for funding the study, and the schools and schoolchildren who participated in the study. We also thank Natalie Richards and Kim Sheppard for administrative assistance, Sophia Lewis for assistance with questionnaire design, and all fieldworkers who assisted with data collection.

CONTRIBUTORSHIP

GM JH and LM were investigators on the CHETS 2 study, and were involved in study conception and design. The set-up of the survey was managed by NA, under the supervision of GM and JH, and the conduct of the survey was managed by NA, under the supervision of GM. GM developed the paper plan, led data analysis and drafting of the manuscript. HL assisted with data analysis and drafting of the manuscript. SL wrote the first draft of a

For peer review only

literature review which informed the background section. All authors contributed to drafts of the full manuscript and approved the final draft.

FUNDING

The lead author is supported by an MRC Population Health Scientist Fellowship (MR/K021400/1). The study was funded by the Public Health Division, Welsh Government. The work was undertaken with the support of The Centre for the Development and Evaluation of Complex Interventions for Public Health Improvement (DECIPHer), a UKCRC Public Health Research Centre of Excellence. Joint funding (MR/KO232331/1) from the British Heart Foundation, Cancer Research UK, Economic and Social Research Council, Medical Research Council, the Welsh Government and the Wellcome Trust, under the auspices of the UK Clinical Research Collaboration, is gratefully acknowledged.

DATA SHARING STATEMENT

No additional data available.

COMPETING INTERESTS

The authors declare that they have no competing interests.

KEY MESSAGES

What is already known on this subject

- Many countries worldwide have introduced legislation banning smoking in public places.
- Short-term evaluations show that legislation was followed by small declines in childhood exposure to secondhand smoke.
- In many countries, increases in voluntary restrictions on smoking in cars and homes were observed after legislation.

For peer review only

- Many children, particularly from poorer backgrounds, continued to report that smoking is allowed in cars and homes.

What important gaps in knowledge exist on this topic

- Little data is available on whether the de-normalisation of smoking in cars and homes observed after smoke-free legislation continued in the longer term.

What this study adds

- Seven years after implementation of smoke-free legislation in Wales, the percentage of children reporting that smoking is allowed in their family cars has halved, while smoking in the home has declined substantially.
- However, among children with parents who smoke, 1 in 5 continue to allow smoking in their car, while almost half continue to smoke in the home.
- Although declining across the socioeconomic spectrum, children from poorer families remain most likely to report that smoking is allowed in their car or home.

REFERENCES

1. Department of Health and Committee on the Medical effects of Air Pollutants. Handbook on air pollution and health. London: The Stationery Office, 1997.

2. Scientific Committee on Tobacco and Health. Update of evidence of health effects of secondhand smoke. London: Department of Health, 2004.

3. World Health Organisation. WHO Framework Convention on Tobacco Control. Geneva: WHO Document Production Services, 2005.

4. Hyland A, Barnoya J, Corral JE. Smoke-free air policies: past, present and future. Tobacco Control 2012;**21**(2):154-61.

For peer review only

5. Öberg M, Jaakkola MS, Woodward A, et al. Worldwide burden of disease from exposure to second-hand smoke: a retrospective analysis of data from 192 countries. The Lancet 2011;**377**(9760):139-46.

6. Bearer CF. Environmental health hazards: how children are different from adults. The Future of Children 1995:11-26.

7. Ho SY, Wang MP, Lo WS, et al. Comprehensive smoke-free legislation and displacement of smoking into the homes of young children in Hong Kong. Tobacco Control 2010;**19**(2):129-33.

8. Adda J, Cornaglia FJ. The effect of taxes and bans on passive smoking. IZA Discussion Paper No. 2191. 2006. Available at SSRN: http://ssrn.com/abstract=919963.

9. Akhtar PC, Haw SJ, Currie DB, et al. Smoking restrictions in the home and secondhand smoke exposure among primary schoolchildren before and after introduction of the Scottish smoke-free legislation. Tobacco control 2009;**18**(5):409-15.

10. Phillips R, Amos A, Ritchie D, et al. Smoking in the home after the smoke-free legislation in Scotland: qualitative study. bmj 2007;**335**(7619):553.

11. Jarvis MJ, Sims M, Gilmore A, et al. Impact of smoke-free legislation on children's exposure to secondhand smoke: cotinine data from the Health Survey for England. Tobacco Control 2011.

12. Moore GF, Currie D, Gilmore G, et al. Socioeconomic inequalities in childhood exposure to secondhand smoke before and after smoke-free legislation in three UK countries. Journal of Public Health 2012;**34**(4):599-608.

13. Moore GF, Holliday JC, Moore LAR. Socioeconomic patterning in changes in child exposure to secondhand smoke after implementation of smoke-free legislation in Wales. Nicotine & Tobacco Research 2011;**13**(10):903-10.

For peer review only

14. Mons U, Nagelhout GE, Allwright S, et al. Impact of national smoke-free legislation on home smoking bans: findings from the International Tobacco Control Policy Evaluation Project Europe Surveys. Tobacco Control 2013;**22**(e1):e2-e9.

15. Cheng K-W, Glantz SA, Lightwood JM. Association between smokefree laws and voluntary smokefree-home rules. American journal of preventive medicine 2011;**41**(6):566-72.

16. Akhtar PC, Currie DB, Currie CE, et al. Changes in child exposure to environmental tobacco smoke (CHETS) study after implementation of smoke-free legislation in Scotland: national cross sectional survey. BMJ 2007;**335**(7619):545.

17. Holliday J, Moore G, Moore L. Changes in child exposure to secondhand smoke after implementation of smoke-free legislation in Wales: a repeated cross-sectional study. BMC Public Health 2009;**9**(1):430.

18. Freeman B, Chapman S, Storey P. Banning smoking in cars carrying children: an analytical history of a public health advocacy campaign. Australian and New Zealand journal of public health 2008;**32**(1):60-65.

19. Baxi R, Sharma M, Roseby R, et al. Family and carer smoking control programmes for reducing children's exposure to environmental tobacco smoke. Cochrane Database of Systematic Reviews 2014(3):CD001746

20. Semple S, Apsley A, Galea KS, et al. Secondhand smoke in cars: assessing children's potential exposure during typical journey conditions. Tobacco Control 2012;**21**(6):578-83.

21. Murphy-Hoefer R, Madden P, Maines D, et al. Peer Reviewed: Prevalence of Smoke-Free Car and Home Rules in Maine Before and After Passage of a Smoke-Free Vehicle Law, 2007–2010. Preventing chronic disease 2014;**11**.

22. ASH Scotland. Secondhand smoke in cars. Edinburgh, 2011.

For peer review only

23. Roberts. C., Kawol. K. Smoking in Cars Carrying Children: monitoring public attitudes, November 2013 update, 2014.

24. Thomson G, Wilson N. Public attitudes to laws for smoke-free private vehicles: a brief review. Tobacco control 2009;**18**(4):256-61.

25. British Medical Association Board of Science. Smoking in vehicles: A briefing from the Board of Science: British Medical Association,, 2011.

26. Hopkinson NS, Majeed A, Britton J, et al. Respiratory health professionals call on MPs to vote to ban smoking in cars with children. BMJ: British Medical Journal 2014;**348**.

27. Dolcini MM, Adler NE, Lee P, et al. An assessment of the validity of adolescent self-reported smoking using three biological indicators. Nicotine & Tobacco Research 2003;**5**(4):473-83.

28. Department of Health. Drug use, smoking and drinking among young people in England in 2003. 2004;**[Internet] Available from http://www.dh.gov.uk/assetRoot/04/09/89/17/04098917.pdf [Accessed 25/01/05]**.

29. Currie C, Molcho M, Boyce W, et al. Researching health inequalities in adolescents: The development of the Health Behaviour in School-Aged Children (HBSC) Family Affluence Scale. Social Science & Medicine 2008;**66**(6):1429-36.

30. Healey B, Hoek J, Wilson N, et al. Youth exposure to in-vehicle second-hand smoke and their smoking behaviours: trends and associations in repeated national surveys (2006–2012). Tobacco Control 2013.

31. Rowa-Dewar N, Amos A, Cunningham-Burley S. Children's Perspectives on How Parents Protect Them From Secondhand Smoke in Their Homes and Cars in Socioeconomically Contrasting Communities: A Qualitative Study. Nicotine & Tobacco Research 2014;**16**(11):1429-35.

For peer review only

32. Hitchman SC, Fong GT, Zanna MP, et al. Support and correlates of support for banning smoking in cars with children: findings from the ITC Four Country Survey. The European Journal of Public Health 2011;**21**(3):360-65.

For peer review only

STROBE Statement—checklist of items that should be included in reports of observational studies

	Item No	Recommendation
**Title and abstract**	1	(*a*) Indicate the study's design with a commonly used term in the title or the abstract. x
		(*b*) Provide in the abstract an informative and balanced summary of what was done and what was found. x
**Introduction**		
Background/rationale	2	Explain the scientific background and rationale for the investigation being reported. x
Objectives	3	State specific objectives, including any prespecified hypotheses. x
**Methods**		
Study design	4	Present key elements of study design early in the paper x
Setting	5	Describe the setting, locations, and relevant dates, including periods of recruitment, exposure, follow-up, and data collection x
Participants	6	(*a*) *Cohort study*—Give the eligibility criteria, and the sources and methods of selection of participants. Describe methods of follow-up<br>*Case-control study*—Give the eligibility criteria, and the sources and methods of case ascertainment and control selection. Give the rationale for the choice of cases and controls<br>*Cross-sectional study*—Give the eligibility criteria, and the sources and methods of selection of participants x
		(*b*) *Cohort study*—For matched studies, give matching criteria and number of exposed and unexposed<br>*Case-control study*—For matched studies, give matching criteria and the number of controls per case
Variables	7	Clearly define all outcomes, exposures, predictors, potential confounders, and effect modifiers. Give diagnostic criteria, if applicable x
Data sources/ measurement	8*	For each variable of interest, give sources of data and details of methods of assessment (measurement). Describe comparability of assessment methods if there is more than one group x
Bias	9	Describe any efforts to address potential sources of bias x
Study size	10	Explain how the study size was arrived at x
Quantitative variables	11	Explain how quantitative variables were handled in the analyses. If applicable, describe which groupings were chosen and why x
Statistical methods	12	(*a*) Describe all statistical methods, including those used to control for confounding x
		(*b*) Describe any methods used to examine subgroups and interactions x
		(*c*) Explain how missing data were addressed x
		(*d*) *Cohort study*—If applicable, explain how loss to follow-up was addressed<br>*Case-control study*—If applicable, explain how matching of cases and controls was addressed<br>*Cross-sectional study*—If applicable, describe analytical methods taking account of sampling strategy x
		(*e*) Describe any sensitivity analyses

Continued on next page

For peer review only - http://bmjopen.bmj.com/site/about/guidelines.xhtml

**Results**		
Participants	13*	(a) Report numbers of individuals at each stage of study—eg numbers potentially eligible, examined for eligibility, confirmed eligible, included in the study, completing follow-up, and analysed x
		(b) Give reasons for non-participation at each stage x
		(c) Consider use of a flow diagram n/a
Descriptive data	14*	(a) Give characteristics of study participants (eg demographic, clinical, social) and information on exposures and potential confounders x
		(b) Indicate number of participants with missing data for each variable of interest x
		(c) *Cohort study*—Summarise follow-up time (eg, average and total amount) n/a
Outcome data	15*	*Cohort study*—Report numbers of outcome events or summary measures over time
		Case-control study—Report numbers in each exposure category, or summary measures of exposure
		Cross-sectional study—Report numbers of outcome events or summary measures x
Main results	16	(*a*) Give unadjusted estimates and, if applicable, confounder-adjusted estimates and their precision (eg, 95% confidence interval). Make clear which confounders were adjusted for and why they were included x
		(*b*) Report category boundaries when continuous variables were categorized x
		(*c*) If relevant, consider translating estimates of relative risk into absolute risk for a meaningful time period n/a
Other analyses	17	Report other analyses done—eg analyses of subgroups and interactions, and sensitivity analyses x
**Discussion**		
Key results	18	Summarise key results with reference to study objectives x
Limitations	19	Discuss limitations of the study, taking into account sources of potential bias or imprecision. Discuss both direction and magnitude of any potential bias x
Interpretation	20	Give a cautious overall interpretation of results considering objectives, limitations, multiplicity of analyses, results from similar studies, and other relevant evidence x
Generalisability	21	Discuss the generalisability (external validity) of the study results x
**Other information**		
Funding	22	Give the source of funding and the role of the funders for the present study and, if applicable, for the original study on which the present article is based x

*Give information separately for cases and controls in case-control studies and, if applicable, for exposed and unexposed groups in cohort and cross-sectional studies.

Note: An Explanation and Elaboration article discusses each checklist item and gives methodological background and published examples of transparent reporting. The STROBE checklist is best used in conjunction with this article (freely available on the Web sites of PLoS Medicine at http://www.plosmedicine.org/, Annals of Internal Medicine at http://www.annals.org/, and Epidemiology at http://www.epidem.com/). Information on the STROBE Initiative is available at www.strobe-statement.org.

For peer review only
